# Nanotechnology against the novel coronavirus (severe acute respiratory syndrome coronavirus 2): diagnosis, treatment, therapy and future perspectives

**DOI:** 10.2217/nnm-2020-0441

**Published:** 2021-03-08

**Authors:** Hamid Rashidzadeh, Hossein Danafar, Hossein Rahimi, Faezeh Mozafari, Marziyeh Salehiabar, Mohammad Amin Rahmati, Samaneh Rahamooz-Haghighi, Navid Mousazadeh, Ali Mohammadi, Yavuz Nuri Ertas, Ali Ramazani, Irada Huseynova, Rovshan Khalilov, Soodabeh Davaran, Thomas J Webster, Taras Kavetskyy, Aziz Eftekhari, Hamed Nosrati, Mehdi Mirsaeidi

**Affiliations:** ^1^Cancer Gene Therapy Research Center, Zanjan University of Medical Sciences, Zanjan, Iran; ^2^Zanjan Pharmaceutical Biotechnology Research Center, Zanjan University of Medical Sciences, Zanjan, Iran; ^3^Joint Ukraine-Azerbaijan International Research & Education Center of Nanobiotechnology & Functional Nanosystems, Drohobych, Ukraine, Baku, Azerbaijan; ^4^Drug Applied Research Center, Tabriz University of Medical Sciences, Tabriz 51656-65811, Iran; ^5^Department of Plant Production & Genetics, Faculty of Agriculture, University of Zanjan, Zanjan, Iran; ^6^Department of Biomedical Engineering, Erciyes University, Kayseri 38039, Turkey; ^7^ERNAM-Nanotechnology Research & Application Center, Erciyes University, Kayseri 38039, Turkey; ^8^Institute of Molecular Biology & Biotechnologies, Azerbaijan National Academy of Sciences, 11 Izzat Nabiyev, Baku AZ 1073, Azerbaijan; ^9^Department of Biophysics & Biochemistry, Baku State University, Baku, Azerbaijan; ^10^Maragheh University of Medical Sciences, Maragheh 78151-55158, Iran; ^11^Department of Chemical Engineering, Northeastern University, 360 Huntington Avenue, Boston, MA 02115, USA; ^12^Department of Surface Engineering, The John Paul II Catholic University of Lublin, 20-950 Lublin, Poland; ^13^Drohobych Ivan Franko State Pedagogical University, 82100 Drohobych, Ukraine; ^14^Russian Institute for Advanced Study, Moscow State Pedagogical University, 1/1, Malaya Pirogovskaya St, Moscow 119991, Russian Federation; ^15^Polymer Institute of SAS, Dúbravská cesta 9, Bratislava 845 41, Slovakia; ^16^Department of Public Health Sciences, University of Miami, Miami, FL 33146, USA

**Keywords:** coronavirus, COVID-19, diagnosis, nanotechnology, SARS-CoV-2, treatment

## Abstract

Coronavirus 2019 (COVID-19), as an emerging infectious disease, has caused significant mortality and morbidity along with socioeconomic impact. No effective treatment or vaccine has been approved yet for this pandemic disease. Cutting-edge tools, especially nanotechnology, should be strongly considered to tackle this virus. This review aims to propose several strategies to design and fabricate effective diagnostic and therapeutic agents against COVID-19 by the aid of nanotechnology. Polymeric, inorganic self-assembling materials and peptide-based nanoparticles are promising tools for battling COVID-19 as well as its rapid diagnosis. This review summarizes all of the exciting advances nanomaterials are making toward COVID-19 prevention, diagnosis and therapy.

## Lay abstract

The emergence of the new coronavirus 2019 (COVID-19) has affected millions of people around the world and has become a serious threat to human life. To date, nanotechnology-based tools have been effective in preclinical studies against a variety of pathogens, including respiratory viruses, herpes virus, human papillomavirus and HIV. Nanoparticles, particles smaller than a micrometer, have drawn particular interest in the management of COVID-19 disease due to their unique properties (easy preparation, low cost, easy modification, appropriate size, etc). Nanotechnology-based strategies for COVID-19 disease management include the development of tools for rapid, accurate and sensitive diagnosis, production of effective disinfectants, delivery of mRNA vaccines into human cells and delivery of antiviral agents into the body.

The new coronavirus 2019 (COVID-19) outbreak, which emerged in Wuhan, China, has infected millions of people worldwide and has become a global threat [1]. COVID-19 is rapidly spreading, causing the deaths of large numbers of people worldwide, and the death rate has been exponentially increasing [2]. Coronaviruses are a group of enveloped viruses with a single-stranded RNA genome approximately 26–32 kb in size that infect not only humans but also animals, including birds and mammals [3–5]. Rapid mutation, altered tissue tropism, cross-species transmission and adaptation to various epidemiological conditions are the main characteristics of this group of viruses [6]. This group of viruses is the largest group of viruses belonging to the Nidovirales order, Cornidovirineae suborder and Coronaviridae family. The Coronaviridae family includes two subfamilies: Letovirinae (Alphaletovirus) and Orthocoronavirinae (Alphacoronavirus [αCoV], Betacoronavirus [βCoV], Gammacoronavirus [γCoV] and Deltacoronavirus [δCoV]) [7,8]. Human coronaviruses first were identified in the 1960s and so far there have been six types identified. Four types, including OC43, 229E, NL63 and HKU1 cause the common cold and gastrointestinal infections and the other two include the severe acute respiratory syndrome coronavirus (SARS-CoV) and the Middle East respiratory syndrome coronavirus with high morbidity and mortality which have attracted a lot of attention and have caused great concern [9].

The causative agent of COVID-19, named SARS-CoV-2, has a 96.3, 89 and 82% nucleotide similarity to bat CoV RaTG13, SARS-like CoV ZXC21 and SARS-CoV, respectively, confirming its zoonotic origin [10–13]. Generally, physical contact, airborne droplets and fomites are common ways of transmitting respiratory diseases such as COVID-19. The transmission of the infection via physical contact refers to the direct transmission of the infection from an infected person to the next and fomites refer to the indirect transmission of the infection through intermediate objects [14]. Fever, cough and tiredness are the main symptoms and shortness of breath, headache, anorexia, sore throat, vomiting, diarrhea, abdominal pain, panting and rhinorrhea are the less common symptoms of COVID-19. Moreover, the severity of the disease may vary if underlying diseases such as elevated blood pressure, diabetes and coronary heart disease are present [12,15]. A high viral load in the upper respiratory tract and also because many people with COVID-19 are asymptomatic has resulted in high COVID-19 transmission between persons [16].

SARS-CoV-2 contains four structural proteins including spike (S), membrane (M), nucleocapsid (N) and envelope (E) proteins. S protein with a molecular weight of 250 kDa is found on the virus surface and involved in corona formation (Figure 1) [17–19]. SARS-CoV-2 enters via the S protein into the host cells and replicates its genome within the host cells. Actually, the main stage in pathogenesis by SARS-CoV-2 is the entry of SARS-CoV-2 into host cells, which begins by binding to a specific receptor on the surface of host cells and then entering the endosome and eventually fusing the lysosomal and viral membranes [20,21]. Suppressing the activity of the S protein is one way to treat the SARS-CoV-2 disease which can block the virus [22,23]. The N protein possessing a molecular weight of 43–50 KDa is the only protein among the structural proteins of SARS-CoV-2 whose core role is to bind to the virus genome with several amino acids, lysine and arginine, and to form nucleocapsids [24,25]. The transient expression of the N protein has been shown to significantly increase the production of viral-like particles (VLPs), suggesting that it may be involved in the formation of a complete virion rather than a viral envelope [26–28]. The M protein, possessing a molecular weight of 25–35 kDa, is a transmembrane glycoprotein type III and is the most abundant protein on the surface of the virus [29]. This protein is the main organizer of virus assembly and interacts with other structural proteins [29]. Binding M and N proteins together stabilize both the nucleocapsid and the virion’s inner nucleus, which eventually completes the viral assembly [30–32]. Among the structural proteins of SARS-CoV-2, the E protein is the smallest protein with a molecular weight of 10–74 kDa. The E protein is abundantly expressed within infected cells during the proliferation cycle, however, a small proportion is incorporated into the virion envelope [33,34]. In addition to the structural proteins encoding genes, specific regions of the virus genome have been identified which encode proteins required for viral replication, including papain-like protease (PLpro) and coronavirus main protease (3CLpro) [35]. The early diagnosis of the disease would lead to early measures and hence effective management of the disease due to the extremely rapid spreading of COVID-19 between persons. The availability of the entire SARS-CoV-2 genome sequence has enabled researchers to design and develop PCR kits for the detection of COVID-19. Currently, the diagnostic kits used for the diagnosis of COVID-19 are based on the diagnosis of the SARS-CoV-2 nucleic acid (quantitative reverse transcription PCR [RT-qPCR]) [36]. This approach faces challenges such as limited access to the required equipment and reagents, necessity of skilled personnel, potential false negative results and time consuming procedures [37].

**Figure 1.**
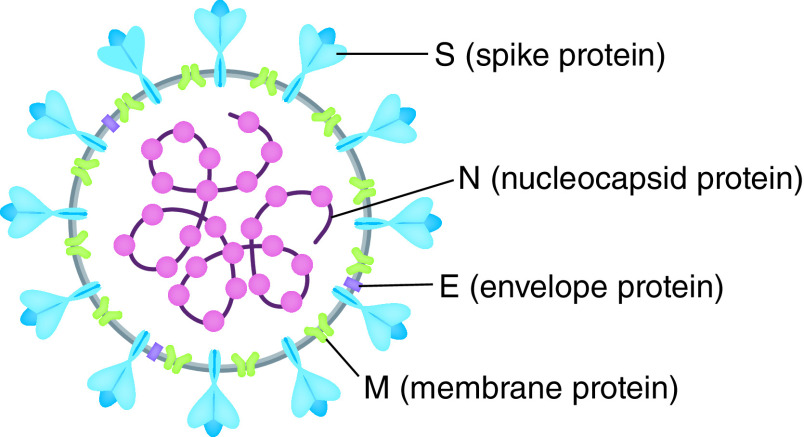
Structure of severe acute respiratory syndrome coronavirus 2. Reproduced with permission from [17] © American Chemical Society (2021).

To date, platforms based on nanotechnology have been effective in preclinical studies toward viral pathogens including such respiratory viruses, herpes simplex, human papillomavirus and HIV [38,39]. Nanotechnology-based techniques can be used to combat COVID-19 pandemics in different ways including development of a sensitive, rapid and specific diagnostic tool for COVID-19, the use of nanomaterials to deliver antiviral agents, improving contact tracing tools, coating of nanomaterial surfaces to inactivate the virus and preparing effective environmental disinfectants [40]. Antiviral agents interfere at certain stages of the virus replication cycle to prevent them from occurring, unlike vaccines which are used to boost the human immune system [41]. Nanomaterials are capable of changing the pharmacokinetic properties of the encapsulated drug and via controlled release mechanism, they can reduce the required concentration of drug. Moreover, the antiviral effects of the established nano-drug can be improved by binding a specific ligand to the surface of the nanoparticle containing the drug for recognition of molecular components of the target tissue/organ [42].

Surprisingly, nanomaterials can create effective interactions between the analyte and the sensor owing to their large surface-to-volume ratios, allowing fast and accurate virus detection [43]. Nanotechnology can improve targeting virus molecules on biological fluids such as nasal, throat and blood samples. Specific virus receptors can be decorated on the surface of magnetic nanomaterials. Since viruses are nanoparticles, virus properties may be used to design virus-like structures to provide targeted drug delivery and gene modifications.

There are concerns such as cargo degradation, no bioavailability or rapid clearance with regard to the delivery of drugs, genes and proteins to the patient’s body, which nanomedicine can address by providing nanoparticle based carriers [44,45]. Different kinds of nanomaterials (such as inorganic, lipid and polymer based nanoparticles) with high loading capacity and unique properties can be used to encapsulate and deliver protein cargos efficiently [46,47]. Intranasal delivery of polymer encapsulated antigens causes a strong immune response and vaccination success relies on polymer type used in combination with the antigen [48,49]. It has been proposed that lipid-based nanostructures could be used to deliver mRNAs or siRNAs to generate viral proteins or vaccination or to deactivate viral target genes [50].

Nanotechnology can also improve the vaccine development. Currently, the most promising vaccines for COVID-19 are made of mRNA from surface proteins of SARS-CoV-2 and encapsulated in nanoliposomes with specific physicochemical properties [51]. Since COVID-19 is transmitted through respiratory droplets and direct contact, disinfecting the air, skin or surrounding surfaces are important preventive measures [52]. Despite the effectiveness of chemical disinfectants in disinfecting air and contaminated surfaces, they face problems such as the need for higher concentrations to completely suppress the virus, insufficient efficiency over time and possible threats to public health and the environment [53–55]. Metal nanoparticles such as silver, copper and titanium dioxide are alternatives to currently used chemical disinfectants with unique antiviral activities, durability and efficacy in low concentrations [56]. These metal nanoparticles with photo-dynamic and photo-thermal capabilities can be used for COVID-19.

## Nanotechnology-based treatments for COVID-19 infections

Currently, the US FDA has only approved remdesivir for COVID-19, but no vaccines at this point. Therefore, the development of novel and effective strategies is highly demanded and one promising approach is to mitigate this disease through the use of nanotechnology [57].

It has been suggested that nanotechnology-based vaccines or monoclonal antibodies will be a promising approach for effective treatment and quick diagnosis. The development of novel nano-therapeutic materials for improving the efficacy of treatment of COVID-19 could be based on following strategies: fabrication of polymeric nanoparticles with rapid and high mucus penetration features, development of biodegradable, nontoxic and stable nanoparticles which are intended to be implemented in the lung with minimum pulmonary toxicity during treatment and surface modification of the nanoparticles by conjugation of PEG as a capping agent and targeting moieties to minimize adverse effects and effective therapy.

**Figure 2.**
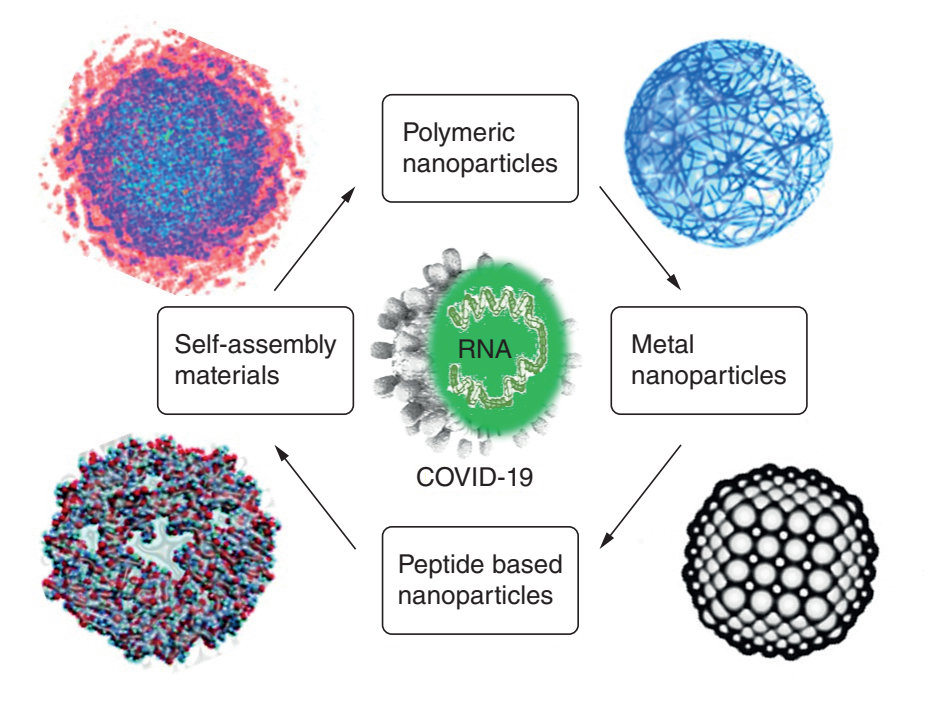
Nanostructured materials in battling coronavirus 2019 and the rapid diagnosis of coronavirus 2019.

A list of nanostructured materials with active and effective antiviral activities is shown in Figure 2.

### Polymeric-based nanostructured materials

**Figure 3.**
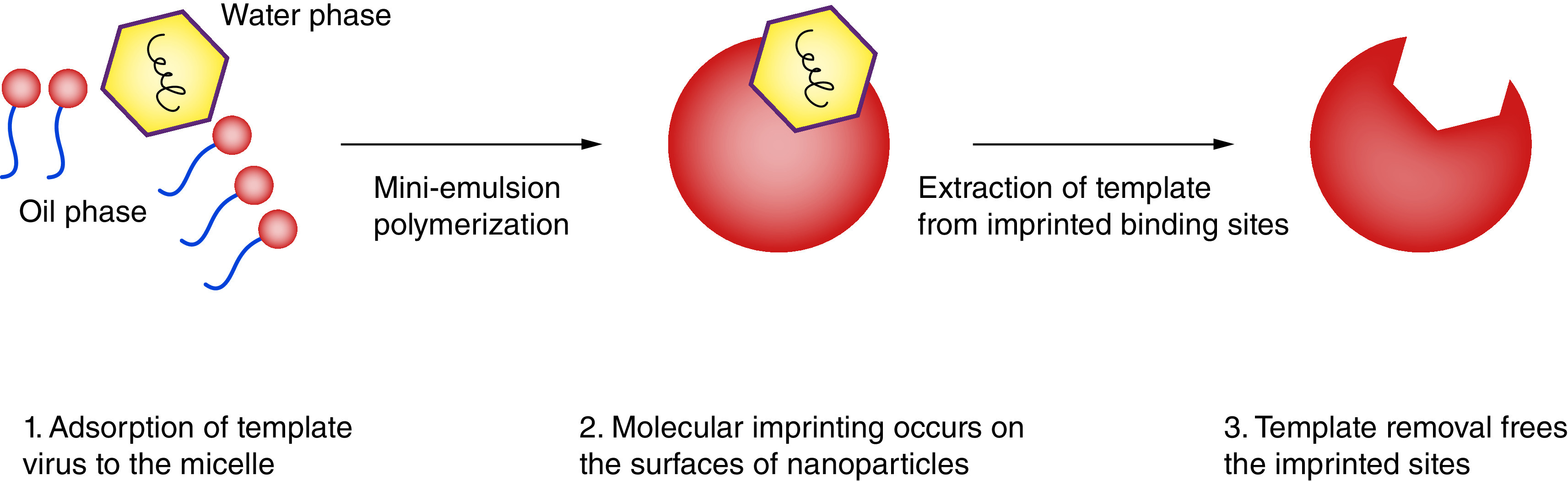
Schematic illustration of polymeric virus catchers fabricated using molecular imprinting techniques. Reproduced with permission from [65] licensed with CC BY 3.0.

Polymeric based nanostructured materials may be a powerful tool in fighting against COVID-19. They have acceptable safety profiles, good biocompatibility and biodegradability, feasible synthetic protocols, prevent degradation of encapsulated drugs and the capability of being simply modified into preferred shapes and sizes and tunable properties [58–60]. Poly (lactic-co-glycolic acid) (PLGA) and PEG are FDA approved polymers which can be employed against COVID-19. This is because of their outstanding biocompatibility and biodegradability when introduced into the human body [61–63]. Although polymeric nanoparticles are useful tools for drug delivery, they are rapidly taken up by the reticuloendothelial system. In order to overcome the aforementioned drawback, surface modification by PEG is needed [64]. For example, Sankarakumar *et al.* developed polymeric virus catchers using molecular imprinting technique as a cost-effective, more stable, rapid and safe mode of anti-viral therapy (Figure 3) [65].

**Figure 4.**
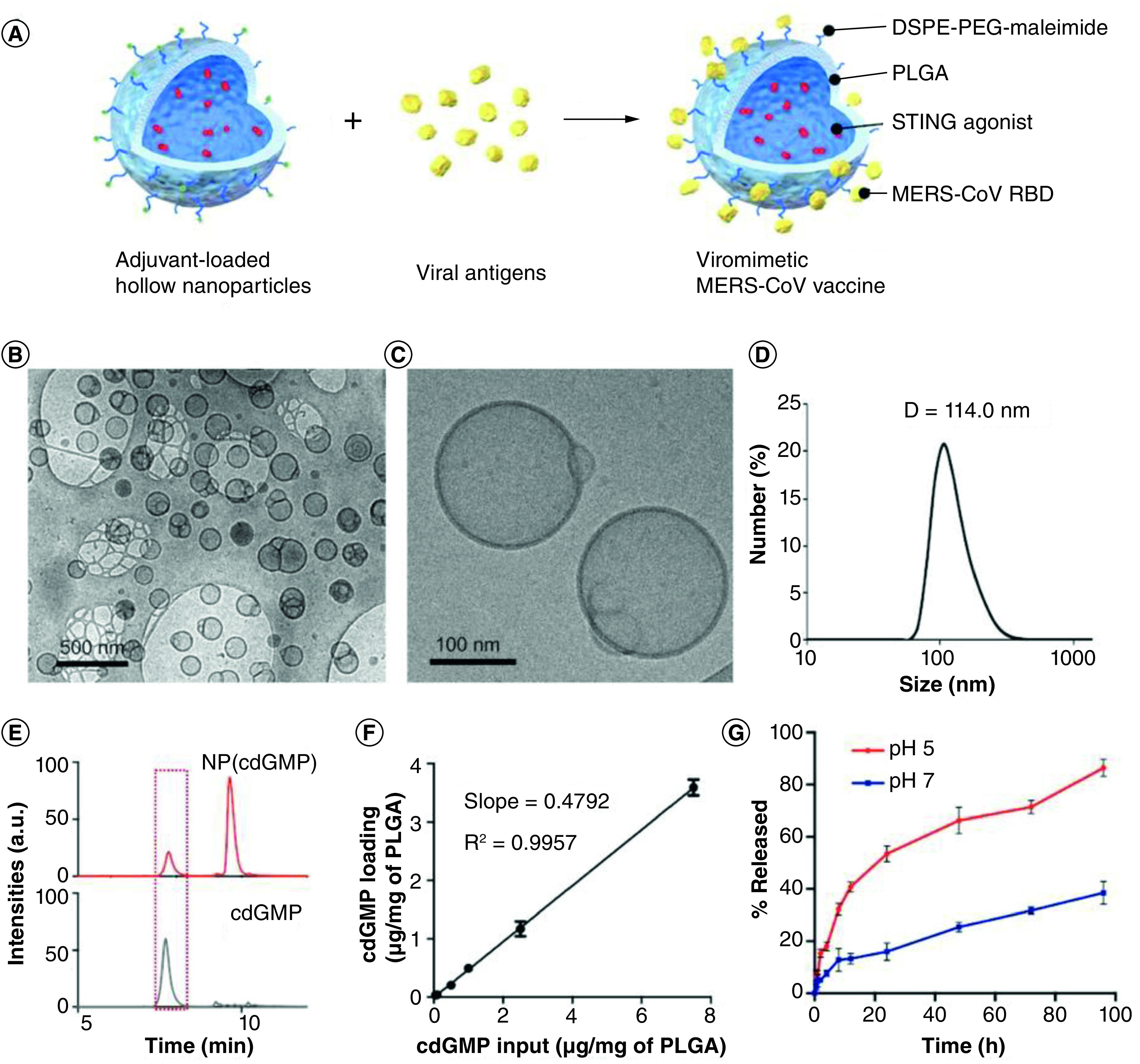
Hollow nanoparticles against coronavirus disease-19. **(A)** Fabrication of a nanovaccine based on synthetic biodegradable PLGA and DEPE-PEG polymers using a double emulsion technique. **(B, C)** Cryo-electron microscopy of cdGMP-loaded hollow nanoparticles. **(D)** Determination of the size distribution of nanoparticles via dynamic light scattering. HPLC diagram of adjuvant-loaded nanoparticles (NP[cdGMP]) and soluble cdGMP. **(E)** HPLC diagram of soluble cdGMP and adjuvant-loaded nanoparticles (NP[cdGMP]). **(F)** Calibration curve for the determination of cdGMP encapsulation efficiency. **(G)** cdGMP release profiles at pH 5 and pH 7. PLGA: Poly (lactic-co-glycolic acid). Reproduced with permission from [66] © WILEY-VCH Verlag GmbH & Co. KGaA, Weinheim (2019).

Lin *et al.* fabricated a novel nanovaccine based on synthetic biodegradable PLGA and DEPE-PEG polymers to deliver subunit viral antigens and STING agonists and an adjuvant in a virus-like fashion as a safe and effective prophylactic measures against Middle East Respiratory Syndrome Coronavirus (Figure 4). The developed capsid-like hollow nanostructured polymer possessed multiple precious and favorable properties such as lowering systemic reactogenecity, pH-responsive release profile and prominent local immune activation. Through the conjugation of antigens, nanoparticles bear a very close resemblance to native virions in morphological properties and co-delivery of both STING agonists and antigens can significantly promote immune potentiation as illustrated in Figure 4. The results indicated that this strategy facilitated an accelerated development of safe and effective vaccines in battling with emerging viral pathogens [66].

### Self-assembling protein based nanoparticles

A novel group of nanoparticles has been produced via oligomerization of monomeric proteins, in which its building blocks were obtained by recombinant technologies, also in the formation of Self-Assembling Proteins based Nanoparticles **(**SANPs) which possess multiple biomedical applications [67]. The production of SANPs in the range of a virus size (20–100 nm) makes them propitious candidates for the generation of a nanovaccine against respiratory viruses [68]. Roux *et al.* developed SANPs based on nucleoprotein (N) from the respiratory syncytial virus (RSV) nucleocapsid and explored it as a potential vaccine antigen in the RSV mouse model. They found that the vaccinated mice were predominantly protected against RSV replication and lower viral load in the lungs. Mucosal immunization with fabricated nanovaccines elicited both systemic and local immunity with high titers of IgA anti-N antibodies, IgG2a and IgG1, antigen-specific CD8^+^ and CD4^+^ T cells [69]. Louis *et al.* modified the nanovaccine by adding the palivizumab-targeted epitope (called FsII) to the N protein and formed a potential nanovaccine with enhanced immune response against RSV. They also found that the load of RSV in the lungs of challenged mice were significantly reduced [70]. VLPs are supramolecular assemblies with diameters ranging from 20 to 200 nm, spherical in shape and generated from the self-assembly of viral capsid proteins. Although VLPs have no viral genetic materials, they can be considered as a potential tool for vaccine production since they have advantages of impeccably mimicking the antigenic epitopes and structure of their corresponding native viruses. Hence, the presence of successive antigens on a particle’s surface (antigen-bearing particles) can promote efficient phagocytosis by antigen-presenting cells and subsequent activation [71,72].

Recently, Lee *et al.* revealed that the administration of VLPs containing multiple ectodomains of matrix protein 2 (M2e5x VLP) of influenza virus by an intranasal route could induce both humoral and cellular immune responses. Intranasal vaccination of the developed vaccine lowered viral loads [73].

### Peptide-based nanoparticles

Studies have reported that mutations of amino acids and short peptide inhibitors (SPI) are promising tools against infections associated with SARS-CoV [74]. The development of vaccines based on peptides which could express the C-terminal heptad repeat region, in a trimeric coil conformation stage, would be an ideal therapeutic approach for the treatment of SARS-CoV associated infections and this strategy is established by employing peptide-based nanoparticles (PBNPs) [75]. More recently, Han and colleagues designed and simulated peptide inhibitors against COVID-19. They prepared peptide inhibitors (PI1–4) extracted from angiotensin-converting enzyme 2 (ACE2) which acts as a highly promising tool to block the COVID-19 receptor binding domains. PI 1 is inclusive of α_1_ (residues 21 to 55), PI 2 is inclusive of α_1_, α_2_ and the loose chain between β_3_ and β_4_ (residues 21–88 and 349–357), PI 3 is inclusive of α_1_, α_2_ and β_3_, β_4_ (residues 21–105 and 323–362) and the composition of PI 4 was similar with PI 3 but different linkage (residues 21–95 and 335– 400). In the initial effort to block COVID-19, SPIs were investigated and amino acid mutations were employed to the S protein of SARS-CoV, but SPIs shortly kept thier secondary structures. For multivalent binding and blocking of the COVID-19 receptors, successive peptides should be conjugated to the surfaces of nanostructured materials. It is achievable by utilizing the PBNPs which potentially neutralized COVID-19 infections [76]. Recently, an SPI extracted from ACE2 provided noteworthy traces for blocking of COVID-19. Additionally, it was demonstrated that the binding efficacy can be promoted by providing multiple binding of SPI onto the nanocarriers [77,78].

### Inorganic & metal nanoparticles

Inorganic nanoparticles exhibit a broad range of applications in the medical filed [79,80]. Their attractive features such as biocompatibility, ease of synthesis, controllable size and unique optical and physiochemical properties make them suitable for biological applications. This group of nanoparticles include a complex hybrid of materials in which its core is made of inorganic agents while its outer shell is composed of organic moieties [81,82]. Among the inorganic nanoparticles, gold nanoparticles (AuNPs) are ideal for vaccine development, because these nanoparticles are readily internalized via both dendritic cells and macrophages, leading to their activation [83,84].

The production of AuNPs is possible because of their easy production in large-scale and with desired particle sizes, they can also be readily functionalized due to the strong affinity between thiol groups and gold [85–87]. Additionally, inert carriers, like AuNPs, do not elicite any immune response [88]. Hence, AuNPs are an appealing platform for the development of nanovaccines by antigen functionalization [89]. Several moieties, such as antigens and adjuvants, can be attached at high density on AuNPs, leading to enhanced antigen presentation and immunogenicity [90,91]. These nanoparticles can be employed for intranasal administration as they can be carried into the lymph nodes, resulting in triggering a robust antigen specific cytotoxic T-cell immune response [92,93]. For example, Sekimukai *et al.* evaluated two types of vaccine adjuvants, based on Toll-like receptor (TLR) agonists and AuNPs, against COVID-19 infections [94]. These two adjuvants already have proven their roles in vaccine engineering and AuNPs, and the antigen carriers as agonists of TLR have been used for developing an ultraviolet-inactivated SARS-CoV vaccine.

**Figure 5.**
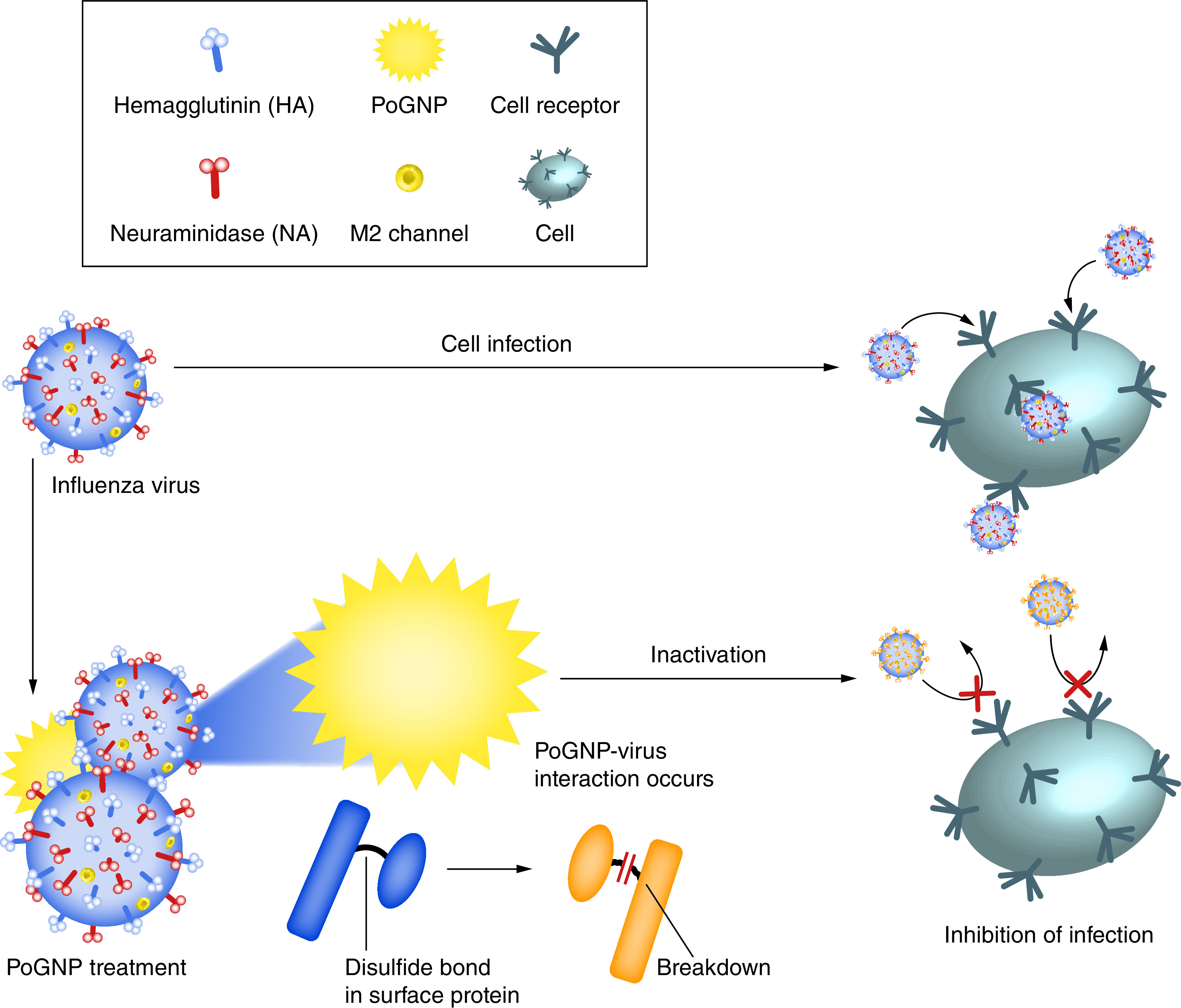
Inactivation of influenza A virus treated with porous gold nanoparticles. PoGNP interacts with Influenza A virus surface proteins and cleaves their disulfide bonds. Inactivated viruses exhibit lower infectivity to cells. PoGNP: Porous gold nanoparticle. Reproduced with permission from [96] licensed with CC BY 4.0.

The AuNP-adjuvanted protein could induce a strong IgG response, but causes a highly allergic inflammatory response. However, a TLR agonist-adjuvanted vaccine could successfully induce protective antibodies without a Th1/17 cytokine response and eosinophilic infiltration. Li and colleagues developed a novel subunit vaccine based on AuNPs and the E2 protein against the recombinant classical swine fever virus E2 protein (CSFV E2). The E2 protein was successfully conjugated to AuNPs to generate stable particle complexes called E2–AuNPs. According to their *in vitro* results, the E2–AuNPs complex exhibited the same immunogenicity as the E2 protein, also AuNPs can enhance the phagocytosis of E2 proteins via antigen-presenting cells. In addition, an *in vivo* study showed that the IFN-γ and IL-10 cytokines, lymphocyte proliferation index and titer of antibody induced by the developed E2–AuNPs were relatively high in comparison with AuNPs or the E2 group. Their findings indicated the potential of employing AuNPs as a vehicle to improve the body’s immune response for fabricating subunit vaccines against CSFV E2. Also this platform can be applied to the development of other flavivirus subunit vaccines like the bovine viral diarrhea virus and hepatitis C virus [95]. Kim *et al.* developed porous gold nanoparticles (PoGNPs) to bind disulfide bonds through gold–thiol interactions against Influenza A virus (Figure 5). The fabricated PoGNPs could predominantly reduce viral infectivity of various Influenza virus strains (H1N1, H3N2 and H9N2). Furthermore, reverse transcription polymerase chain reaction results confirmed that the PoGNPs hampered the fusion of the viral membrane via blocking the entry route of virus through deformation in the conformational of hemagglutinin [96].

## Role of nanotechnology in vaccine development

Generally, nanovaccines are a new generation of vaccines that use nanoparticles to deliver antigens into the human body. Since nanoparticles are in the same size scale as the viral particles, they could effectively enter into the cell and lead to the expression of antigens from the delivered gene construct (DNA or mRNA vaccines) or directly target the immune cells for the delivery of antigens. Through strategies such as oral and intranasal, as well as intramuscular and subcutaneous injections, nanoparticles can be administered to bypass tissue barriers and can target key areas such as lymph nodes [97–100]. Humoral and cellular immunity have been shown to play a protective role in SARS CoV infection [101,102]. Nanoparticles are capable of targeting both adaptive (T cells, B cells) and innate (macrophages, monocytes and neutrophils) immune systems at the cellular level. The ability to deliver molecular adjuvants, as well as possessing intrinsic adjuvant property in some cases for the loaded antigens, is one of the advantages of nanoparticles in increasing the efficacy and safety of the vaccine. Adjuvants enable the cell to easily recognize and respond to antigens by catalyzing the immune response. Physicochemical properties of antigen, target areas, biological stability and the amount of required immunogen release are factors involved in antigen loading inside or on the nanoparticle surface. The physical loading of antigens on nanomaterials relies on its surface charge as well as noncovalent hydrophobic interactions [103,104]. Currently, many vaccine companies encapsulate the gene material, protein/peptide of the vaccine in nanoparticles such as lipid nanoparticles (LNPs). For example, COVID-19 mRNA-based vaccines made by BioNTech/Pfizer and Moderna are encapsulated in LNPs. The mRNA vaccine encapsulated in positively charged LNPs is highly stable and resistant to RNase-mediated degradation and forms self-assembled particles that can be injected in various routs. LNPs are synthesized by self-assembly of an ionizable cationic lipid and due to their similar size to viruses (80–200 nm), they can efficiently deliver mRNA into the cytoplasm [105,106]. Modifications of these nanoparticles with cholesterol or PEG, different LNPs can be developed. It is worth to mention that cholesterol and the PEG-lipid composition could increase stability and half-life of the formulation, respectively. By entry of the LNPs-mRNA vaccine complex into the cell through endocytosis mechanism, endosomal escape is facilitated due to the presence of lipid in composition of LNPs and then it releases mRNA into the cytoplasm, where the mRNA is translated into antigenic proteins that stimulate the immune system which results in the formation of neutralizing antibodies [107–109]. In addition to LNPs, several nanoparticles have been utilized as propitious vehicles for mRNA delivery, such as cationic polymer nanoparticles, polyethyleneimine, oil-inwater (O/W) cationic nanoemulsion and PEG-lipid functionalized dendrimer nanoparticles [103,110].

## Nanomaterial applications in diagnosing COVID-19

Among the above mentioned nanostructured materials, most metallic nanoparticles are intended to be utilized for diagnostic purposes rather than therapy. An accurate, rapid and early diagnosis of COVID-19 is critically important as it can: improve the therapeutic efficacy and effectiveness of the treatment resulting in the avoidance of long-term complications for patients, ascertain areas with increased incidence of infections and help public health officials to monitor the spread of the virus and prognosticate what surge capacity would be needed in infected areas [111]. The WHO and other organizations have concluded that a rapid diagnostic tool is needed in order to end this pandemic. Current screening and diagnosing tests for COVID-19 infection is based on chest computed tomography and rtPCR, respectively [112,113]. Moitra *et al.* fabricated the selective naked-eye detection of COVID-19 based on AuNPs capped with antisense oligonucleotides which can diagnose and detect the number of positive COVID-19 cases from isolated RNA specimens within just 10 min without requiring any advanced instrumental facilities and equipment. The thiol modified antisense oligonucleotides capped AuNPs through gold–thiol interactions could agglomerate selectively in the presence of the corresponding RNA sequence of COVID-19 and then its surface plasmon resonance would undergo changes. Further, by introducing RNaseH, the RNA strand is liberated from the RNA-DNA hybrid, resulting in a visually detectable precipitate [114].

Zhu and colleagues successfully developed a one-step reverse transcription loop-mediated isothermal amplification (RT-LAMP) coupled with nanoparticle-based biosensors (NBS) (RT-LAMP-NBS) for accurate and rapid diagnosing of COVID-19 in a single tube reaction. The sensitivity of the fabricated diagnostic system was 12 copies per reaction and without any generation of any cross-reactivity from non-COVID-19 templates. For patients diagnosed by COVID-19, the sensitivity of the developed RT-LAMP-NBS was 100% (33/33) in oropharynx swab samples and its specificity for non-COVID-19 patients was also 100% (96/96). All steps from the sample collection up to the interpretation of the final test result takes only 1 h [115]. Furthermore, nanoparticle-based tools against respiratory viruses are summarized in Table 1.

**Table 1.** Nanoparticle-based tools against respiratory viruses.

Types of substances used for fabrication	Types of virus	Antigen/epitope	Adjuvant	Mean diameter (nm)	Achievements	Ref.
***Polymeric-based nanostructured materials***						
Polyanhydride	RSV	G and F glycoproteins	–	200–800	Vaccinated mice were largely protected against virus replication in the lungs	[69,116]
HPMA/NIPAM^†^	RSV	F protein	TLR-7/8 agonist	12–25	Nanoparticles displayed appropriate antigenicity and elicited high titers of prefusion-specific, TH1 isotype anti-RSV F antibodies following vaccination	[117,118]
Chitosan	IF(H1N1)	Antigen M2e	Heat shock protein 70°C	200–250	Nanoparticles improved pharmacokinetic profile and enhanced vaccine immunogenicity for inducing antibodies and T cell immunity	[119]
	Swine IF (H1N2)	Killed Swine IF	–	571.7	Intranasal immunization of mice with fabricated nanoparticles directed immune response to a more effective quality profile	[120]
	IF(H1N1)	HA-Split	–	300–350	Nanovaccine elicited strong cross-reactive mucosal IgA and cellular immune responses in the respiratory tract that resulted in a reduced lung virus titers and nasal viral shedding	[121]
	IF(H1N1)	Antigen of H1N1	–	140	Nanovaccine reduced morbidity and conferred 100% protective rate to the vaccinated mice against lethal IF virus	[122]
	IF(H1N1)	Hemagglutinin	–	100–200	After three nasal immunizations, the developed nanovaccine induced significantly high levels of serum IgG and mucosal sIgA	[123]
PLGA^‡^	Swine IF (H1N2)	Inactivated H1N2 virus	–	200–300	Subcutaneous immunization with fabricated vaccine increased the protective immune responses against IF virus in mice	[124]
	BPI3V	BPI3V Proteins	–	225.4	Nanovaccine reduced lung pathology and viral antigenic load in the lung sections with clearance of infectious challenge virus in most of the vaccinated pig lung airways	[125]
***Metal nanoparticles***						
AU	IF	Antigen M2e	CpG	12	Intranasal vaccination of mice with M2e–AuNP conjugates induced M2e-specific IgG serum antibodies and mice vaccinated with soluble CpG as adjuvant in addition to M2e-AuNP were fully protected	[126]
***Self-assembly proteins and peptide-based nanoparticles***						
Q11 peptide^§^	IF(H1N1)	Antigen M2e	–	15–100	Vaccination with developed nanoparticles did not only protect mice against homologous challenge of IF PR8 H1N1 virus, but also provide protection against heterologous challenge of highly pathogenic avian IF H7N9 virus	[127]
Q11, PAQ11^¶^ and SIINFEKLQ11^#^	IF(H1N1)	CD8^+^ epitope	–	–	Intranasally delivered peptide nanofibers were found to be more immunogenic incomparision to subcutaneous route, thereby it produced greater CD8^+^ T cell responses in lung-draining lymph nodes, greater numbers of tissue resident T cells and a more rapid tissue resident memory response to IF infection	[128]
N of RSV	IF(H1N1)	Antigen M2e	Montanide™ Gel01	15	Intranasal vaccination of nanoparticles induces robust immune responses, including high titers of sera M2e-specific IgG antibodies, T-cell immune responses and mucosal secretory-IgA antibodies in mice	[129]
	IF(H1N1)	Antigen M2e	–	10–100	Vaccinated mice presented a reduced viral load and minor weight loss and all survived upon challenge with IF virus	[130]
	RSV	FsII	Montanide™ Gel01	–	Intranasal N-FsII immunization elicited anti-F antibodies in mice that were non-neutralizing *in vitro* and it provided better protection against virus replication especially in the upper airways	[70]
***Other nanoparticles***						
VLP	RSV	M1 protein of IF and RSV-F or -G	–	40–100	Intramuscular VLPs vaccination reduced lung viral load and elicited IgG2a dominant RSV-specific IgG antibody responses against RSV-A2 viruses in both serum and lung extract	[131]
	IF (H1N1, H3N2, H5N1)	Antigen M2e F protein et G	–	60–80	Mice vaccinated intranasally with VLPs showed viral-specific antibody responses against RSV as well as VLPs conferred enhanced protection against live RSV challenges	[132]
	IF(H1N1)	Hemagglutinin	–	80–120	Intranasal vaccination of VLP induced broad cross-protection by M2-specific humoral and cellular immune responses	[73]
Liposome	IF(H1N1)	M2, HA, NP	MPL and trehalose 6,6 dimycolate	10–1000	Nanoparticles elicited virus-specific memory T-cell responses but not neutralizing antibodies	[133]

† N-(2-hydroxypropyl) methacrylamide/N-isopropylacrylamide.

‡ poly(lactic-co-glycolic acid).

§ fibrilizing peptide (Ac-QQKFQFQFEQQ-Am).

¶ (H2N-SSLENFRAYV-SGSG-QQKFQFQFEQQ-Am).

# (H2N-SIINFEKL-SGSG-QQKFQFQFEQQ-Am).

AuNP: Gold nanoparticle; RSV: Respiratory syncytial virus; TLR: Toll-like receptor; VLP: Viral-like particle.

## Unsolved concerns

Despite the global efforts to perceive the specific treatment approaches for COVID-19, various concerns still remain unsolved. COVID-19, which is generated from RNA viruses, are prone to genetic recombination and mutation, thus, it will be considered as a serious global health threat in the future. In the most optimistic condition, the first anti-COVID-19 vaccine would be available in the market during 2020–2021, due to the requirements of large-scale production as well as lengthy and strict regulatory affairs, but until then, all efforts must be done to discover a drastic treatment approach so as to face this respiratory infectious diseases. Recently, a great number of efforts have been made by R&D employees at many pharmaceutical industries as well as researchers and scientists on exploring the applicable anti-virus formulation from the old fashioned drugs or newer ones and vaccine development. Overall, the profound role of nanotechnology and nanoscience is undeniable, but these propitious materials could cause severe issues in lung and respiratory systems. The five main patho-biological aspects are as follows: cell toxicity, fibrosis, inflammation, oxidative stress, genotoxicity and immunotoxicity which must be considered when using nanoparticles or associated approaches for treating current and future coronavirus infections.

## Future possible strategies to tackle COVID-19 by means of nanotechnology

The development of effective nanomedicines against COVID-19 is based on the following approaches; capability of nano-vehicles to bypass the conventional limitations of antiviral therapeutics, co-delivery or combination drug therapeutics by means of nano-vehicles, active targeting by the conjugation of (nano) targeting moieties on the surfaces of nano-vehicles, development of nano-biosensors for rapid detection, anti-viral surface coatings via nanomaterials as a major prevention goal, as well as incorporation of nanomaterials with anti-viral properties into facial masks, gloves and other contaminated surfaces, capability of nanomaterials as disinfectant tools to inactivate or kill the pathogenic microorganisms and vaccine development using nanomaterials and chemically alter/(re)engineer drugs to improve the compatibility of drugs with a particular type or class of nano-vehicles – a generic approach for therapeutics candidates with similar physiochemical properties [134,135].

There is a significant genomic match between COVID-19 and other coronaviruses, therefore, it is valuable to revisit these strategies along with nanotechnology to tackle COVID-19 [136]. Some of the antiviral therapeutics and their water solubility, clinical trial phases and log p along with corresponded half-life (t_1/2_) are summarized in Table 2. According to these data, most of the antiviral therapeutics have poor solubility which causes failure in the effective treatment of viral diseases. Insufficient or low bioavailability of these antiviral therapeutics because of their low solubility may cause them to be dropped off the pipeline [137–139]. The limitations related to current antiviral therapies can be solved using nano-vehicle based therapeutics by modifying their pharmacodynamic/pharmacokinetic characteristics resulting in the improvement of drug bioavailability, reduction of dose, reduced toxicity and maintenance of the suppression of viral spread [140].

**Table 2.** Water solubility, clinical trial phases, log P and half-life (t_1/2_) h of existing antiviral molecules against coronavirus 2019.

Drug	Water solubility (mg/ml)	Log P	Half-life (t_1/2_) h	Recommended dosage	Clinical trial phase(s)
***Practically insoluble***					
Fingolimod	0.0069	4.06	144–216	0.5 mg/day	Phase II
Umifenovir	–	∼4.5	17–21	–	Phase IV
Lopinavir	–	4.69	6.9	200 mg/12 h up to 7–10 days	Phase II
Ritonavir	–	3.9	3–5	100 mg/12 h up to 10 days	Phase IV
***Very slightly soluble***					
Baricitinib	0.357	∼1	12.5	2 mg/day up to 14 days	Phase II, III
Ifenprodil	0.105	3.98	Not available	20 and 40 mg ^†^TID	Phase II, III
Remdesivir	0.339	2.1	14, 20	200 mg on day 1 followed by 100 mg on days 2–10	Phase III
Ruxolitinib	0.116	2.48	2.8	10 mg/12 h	Phase II, III
Darunavir	0.0668	1.89	15	400 mg/day up to 5 days	Phase IV
Camostat	0.0626	1.51	2.8–3.7	400 mg/6 h up to 7 days	Phase II
Hydroxychloroquine	0.0175	2.89	537.6	600 mg/day up to 7 days	Phase II
Chloroquine	0.0175	4.63	480–1440	250 mg/day up to 10 days	Phase II
***Slightly soluble***					
Ribavirin	33.20	-2.8	120–170	400 mg/12 h up to 5 days	Phase II
Favipiravir	8.70	0.25	2–5.5	1800 mg/12 h on day 1 followed by 800 mg/12 h up to 7 days	Phase II, III
Galidesivir	7.40	-2.1	Not available	iv. infusion every 12 h up to 7 days	Phase I
^‡^EIDD-2801	5.77	∼-2	Not available	Oral capsul/day up to 5 days	Phase I
Thalidomide	2.55	0.33	5–7	100 mg/day up to 14 days	Phase II
Emtricitabine	2.00	-0.43	10	200 mg/day up to 60 days	Phase II, III
Tenofovir	1.87	1.25	32	300 mg/day up to 60 days	Phase II, III
Oseltamivir	0.686	1.16	1–3	75 mg/12 h up to 14 days	Phase II

† Three-times a day,

‡ molnupiravir,

Note: The aforementioned drugs were used to treat COVID-19 – either alone or in combination with other medication(s).

COVID-19: Coronavirus 2019.

Vaccine development for COVID-19 is based on the following approaches and the main target strategies for successful vaccine fabrication are; averting the ACE2-mediated host uptake, inducing nanobodies against the viral S protein, which can be achieved by means of nanotechnology, inducing an immune response where antigen-loaded nano-vehicles would be a very promising tool, using engineered nanoparticles as vaccine adjuvants to enhance the overall safety and efficacy of the generated immune response, utilizing the intrinsic adjuvanticity of the nanoparticles by activation of inflammasomes which activates the complement system and induces autophagy [141–146].

Combination therapy, a treatment method that is defined as a combination of two or more therapeutic agents, would become a cornerstone to face COVID-19 in the near future. In the absence of approved world-wide treatment against COVID-19, in many countries, instead of using monotherapy, physicians are prescribing multiple medications simultaneously to enhance treatment efficacy as this approach could target crucial pathways in a characteristically synergistic or an additive manner [147]. To this end, nano drug co-delivery systems which can simultaneously load at least two therapeutic agents with not only physicochemical but also pharmacological properties into a propitious nanovehicles could be proposed as an effective alternative option to combat COVID-19. Moreover, can also be considered as an approach that combines the above-mentioned repurposed approaches with other therapies for instance, encapsulation of therapeutic agents into nanovehicles along with surface modification of the drug carrier by targeting moieties.

One of the bigger challenges in the treatment of COVID-19 is the accumulation of therapeutics at nontargeted or unwanted sites. This could be overwhelmed significantly by applying active targeting of nano-vehicles to direct therapeutics to the intended site of action [79,148]. Furthermore, it is possible to target the intracellular and cellular sites and specific organs including cathepsin binding sites, ACE2 expressing cells and domains of viral S protein which are involved in the pathophysiology of SARS-CoV-2. The release of drugs from nanovehicles in a controlled and sustained manner can provide better patient compliance, minimized side effects and reduced dosage amount and frequency and mitigated risk effects of the viral rebounded during viral infection treatments [149].

In order to end this pandemic, diagnostics could play profound roles, as it is crucial to isolate the confirmed cases of COVID-19 as early as possible to prevent the dissemination [150]. Typically, testing kits rely on either the detection of antibodies (via ELISA) or RNA (via PCR, in which the specimen is taken from the throat or nose of the infected patients) associated with the virus. This approach is based on the interactions between the pathogen surface with a complementary detection strand or ligand which is present in the kit [36]. However, these kits are far from perfect due to the following problems; lack of analytical sensitivity, long response times and false-negative results [37]. To this end, nano-sized materials could potentially instigate highly efficient surface interactions between the analyte and the sensor resulting in rapid, accurate and more reliable detection of the virus [43]. Therefore, in order to shorten the response times to ascertain whether an individual is infected or not, as well as to reduce the burden of disease, nanotechnology could provide faster, more precise, simpler and user-friendly platforms without requiring any highly qualified staff or special facilities.

It has been reported that coronaviruses cannot replicate on any non-living surface such as plastic, fabrics, wood, glass and metal surfaces, but they can remain viable or infectious for several hours to days. Several disinfectants, such as sodium hypochlorite (0.1%), hydrogen peroxide (0.5%) or ethanol (62–71%) are effective at destroying coronaviruses, but it is nearly impossible to continuously sanitize a device surface or contaminated surfaces, since they can be recontaminated. Furthermore, these disinfectants are effective at high concentrations, which can cause environmental and health problems due to their high toxicity. Research into solving the aforementioned problems is already underway, also it is believed that one promising approach to end this pandemic is employing an anti-viral surface coating in which the surface is ought to be non-sticking to the pathogen, repealing the pathogen or self-sanitizing. Recent studies revealed that spike (S) glycoprotein is a key surface protein of coronaviruses, mediating the entrance of the virus into human epithelial cells in the respiratory track by interacting with cell surface receptor ACE2. Hence, as stated above, a wiser solution to face this pandemic is development of a surface coating with a relatively low surface energy value which can repel the S glycoprotein.

A smart multi-functional anti-pathogen coating with ‘anti-adhesion’, ‘contact-killing’ and ‘release-killing’ properties has been already introduced into healthcare settings to fight the increasing threat of infectious diseases. Moreover, an antimicrobial coating solution for almost all surfaces with the aid of nano-actives has been fabricated and the literature has supported them as an anti-viral as well. For example, NANOVA HYGIENE+ as an antimicrobial coating was developed by the incorporation of positively charged silver nanoparticles as bioactive nanoparticles dispersed into binder polymers for coating of surfaces like metals, plastics, fabrics, etc. [151]. In addition, introducing titanium dioxide nanoparticles into a polymeric matrix leading to the generation of a photocatalytic coating (light mediated) can kill and destroy pathogens (viruses) on surfaces upon exposure to light by damaging their membrane. Additionally, to further increase the inhibitory effect of respiratory face masks, nano-actives can also be incorporated into their fabric. These nanomaterials exhibit a great potential as an anti-viral coating against coronaviruses due to their unique properties including intrinsic anti-viral properties such as reactive oxygen species (ROS) generation and photo-dynamic and photo-thermal capabilities.

**Figure 6.**
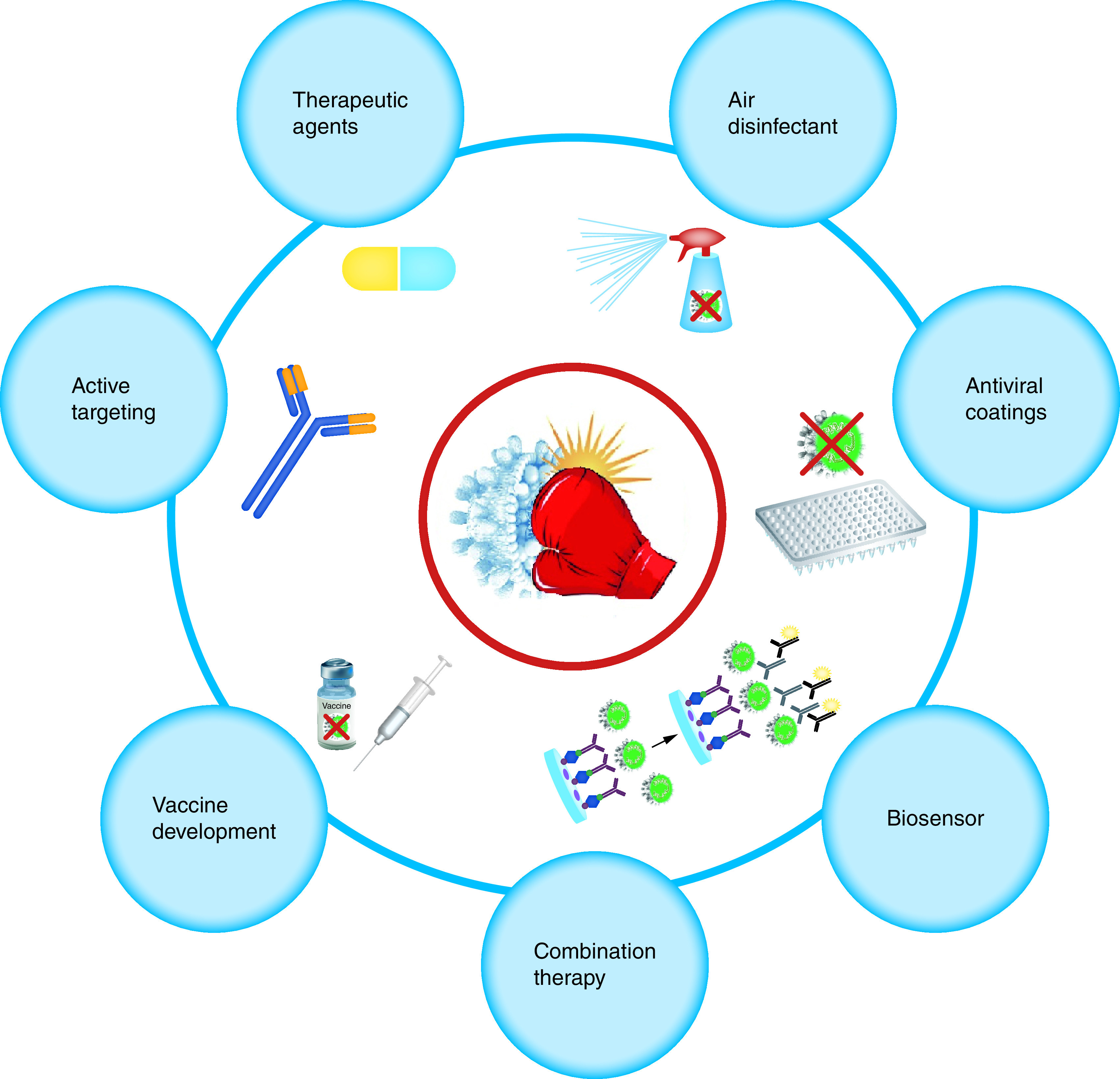
Schematic representation of possible strategies to tackle coronavirus 2019 using nanotechnology.

Coronaviruses can be transmitted through various routes including biofluids, respiratory droplets and coughs [52] and it seems that one auspicious strategy to face this invisible enemy is through preventing its dissemination by routinely applying disinfectants to surrounding surfaces, skin or air. In this regard, different chemical disinfectants such as alcohols, quaternary amines, peroxides and chlorines have been used to tackle the pathogens by means of sterilization and disinfection surfaces and personal protective equipment [53]. Although chemical disinfectants exhibited promising results, their application has been restricted gradually due to several drawbacks such as possible risks to the environment and public health, limited effectiveness over time and 100% viral inhibition is achieved only at high concentration [54,55]. Therefore, inorganic nanoparticles, more specifically metal-based nanomaterials such as titanium dioxide (TiO_2_), copper (Cu) and silver (Ag) nanoparticles, owing to their persistence and ability to be effective at much lower dosages and inherent broad-spectrum antiviral activities, have been proposed as effective alternatives against newly emerging viruses [56,152]. Based on these results, it can be concluded that nanomedicine based strategies are powerful tools to tackle the COVID-19 pandemic. A schematic representation of possible strategies to tackle COVID-19 using nanotechnology is shown in Figure 6.

## Conclusion & future perspective

Global health is confronted with the most dangerous situation regarding the novel SARS-CoV-2, known as the COVID-19 pandemic, affecting people’s life in every region and community in the world. Nanotechnology based medications may play a crucial role in advancing COVID-19 diagnosis, treatment, prevention and vaccine development. Additionally, time is of essence when encountering infectious disease outbreaks and nano-based materials have more of a chance to become available sooner to the public in comparison with other treatment options, since they are not associated with lengthy and strict regulatory affairs that are commonly associated with vaccines. It is necessary to develop broad antiviral spectrum of disinfectants with acceptable efficacy even at low concentrations and shortening response times to ascertain whether an individual is infected or not. Moreover, it is pivotal to overcome problems associated with monotherapy and to reduce the dose of the drug and decrease its adverse effects. Furthermore, development of a surface coating with a relatively low surface energy (which can repel, kill or be anti-adhesive to the pathogens) is a wise solution that can mitigate the burden of diseases at places where the viral load is high. Nano-based materials can take root against the current global public health threat by promoting exactly the type of wide-ranging, integrated approaches that are indispensable to manage and control the COVID-19 outbreak at both the local and international level. Despite the intense studies on the nanotechnology based tools (polymeric, inorganic self-assembling materials, PBNPs and nanovaccine) to mitigate the COVID-19, there have been some drawbacks such as fibrosis, inflammation, oxidative stress, genotoxicity, immunotoxicity, costly, time-consuming, restriction affairs and potential cell toxicity of these nanoparticles, which are not neglectable in rational design and engineering, remain hazy and this make it very difficult to achieve an effective clinical translation. In the not too distant future, we anticipate that many advancements will be achieved in COVID-19 diagnosis, treatment and therapy using nanotechnology based strategies. It is very likely that nanotechnology-based tools will not only be able to be employed in treatment of COVID-19, but also in a wide range of emerging pathogens. This can be achieved using nanotechnology-based vaccines or monoclonal antibodies which precisely deliver the active agents to targeted tissues as well as providing very rapid detection of these viruses. Finally, the biggest challenge will always remain the possibility of these nanomaterials to achieve clinical translation and the feasibility of a scaled-up production.

**Executive summary**

- Recently, the new coronavirus 2019 (COVID-19) outbreak, as an emerging infectious disease was first reported in Wuhan, China, has infected millions of people worldwide and has become a global threat.
- Nanomedicine, as a very power tool, can mitigate the burden of disease by providing nanoparticle-based carriers and vaccines.

**Nanotechnology-based treatments for COVID-19 infections**

- Nano-based therapeutics such as polymeric nanoparticles, self-assembling protein based nanoparticles, peptide-based nanoparticles and inorganic and metallic nanoparticles, owing to their unique features, exhibit a broad range of applications in the medical field, therefore they could be used as promising approaches in COVID-19 treatment.

**Role of nanotechnology in vaccine development**

- The great merits of vaccine development via nanomaterials are their intrinsic adjuvanticity as well as inducing an immune response where antigen-dependent nano-carriers would be a very promising tool.
- Thereby antigens could be loaded whether inside or on the surface of nanocarriers.

**Nanomaterial applications in diagnosing COVID-19**

- Metallic and inorganic nanoparticles due to unique physicochemical characteristics could address critical criteria and considerations for developing clinically translational nanosized devises in the rapid diagnosis of COVID-19.

**Unsolved concerns & perspectives**

- Recombination and mutation could occur in many RNA viruses and it makes the virus more dangerous to humans, also it should be considered as a serious global health threat.
- Nano-based materials, due to their nanosized features, could cause severe problems in lung and respiratory systems and this negative aspect should be considered in rational design of nanoparticles for COVID-19 treatment.

**Future possible strategies to tackle COVID-19 by means of nanotechnology**

- Design and development of effective therapy against COVID-19 is based on the following strategies.
  - Capability of nanomaterials to bypass the conventional restriction associated with antiviral agents.
  - Combination therapeutics using nano-vehicles.
  - Active targeting by decorating specific (nano) targeting moieties on the surface of nanomaterials.
  - Nano-biosensors for rapid detection.
  - Anti-viral surface coatings by the introduction of nanomaterials within the polymer matrix.
  - Capability of nanomaterials as disinfectant agents, and.
  - Vaccine development using nanomaterials and chemically alter/(re)engineer drugs.

**Conclusion & future perspective**

- Fibrosis, inflammation, oxidative stress, genotoxicity, immunotoxicity and potential cell toxicity of these nanoparticles are key issues to be solved before reaching patients.
